# What Is the Current State of Training to Recognise and Treat Eating Disorders in Medical Schools in South Wales?

**DOI:** 10.1192/bjo.2025.10520

**Published:** 2025-06-20

**Authors:** Thomas Prew, Rhiannon Kihara, Umer Jalal, Lois Bown, Isabella Jurewicz

**Affiliations:** 1 Cardiff and Vale University Health Board, Cardiff, United Kingdom; 2 Swansea Bay University Health Board, Swansea, United Kingdom; 3 Cardiff Medical School, Cardiff, United Kingdom; 4 Service for High Risk Eating Disorders, Cardiff, United Kingdom

## Abstract

**Aims:** Eating disorders represent a major challenge for psychiatric and broader medical care. The rate of hospitalisation has almost doubled in recent years and anorexia has a higher mortality rate than any other mental illness. Beat Eating Disorders has made recommendations for educating medical students. When making the recommendations, they did not receive responses from Cardiff or Swansea Medical Schools. This review assesses the provision of eating disorder teaching and whether it is sufficient for effective medical training.

**Methods:** Clinical lecturers in Cardiff and Swansea Medical School reviewed each other’s medical curricula and compared the prevalence and extent of eating disorders in each. From 1 December 2024 to 5 February 2025 we reviewed the types of learning provided, opportunities for self-directed learning, and other areas where eating disorders could arise. We compared this against the guidelines recommended by Beat for an effective curriculum. We also reviewed the schools’ official exam guidelines to assess whether eating disorders are listed as a topic in psychiatry or the broader curriculum.

**Results:** In Cardiff there is a dedicated lecture on eating disorders in the Year 4 psychiatry rotation, which covers all major eating disorders. There is also an optional online module written by an Eating Disorders Consultant which goes into further detail. There is no practical training in examining or communicating with a person with eating disorders.

In Swansea there was no mention of eating disorders in the curriculum yearbook. There is a lecture in Year 2 and Year 3, each an hour long. Eating disorders exist on the GMC MLA content map, so can come up in the final year OSCE (CPSA) but it is not clear whether this happens in practice in Swansea or Cardiff.

Across South Wales, clinical attachments with eating disorder services were haphazard and locality-dependent. Beat would classify both medical schools as providing “insufficient” education.

**Conclusion:** Medical students in Wales are not receiving education on eating disorders that satisfies the Beat recommendations. Despite achieving proficiency in academic teaching, neither medical school provides the practical experience necessary to examine, support, and treat someone with eating disorders.

Greater emphasis on eating disorders is required, not just within psychiatry, but within broader medical teaching such as cardiology and gastroenterology. Eating disorders should be better incorporated into communication stations, practical examinations, and psychiatric teaching. Better access to Eating Disorder Services for medical students would also allow them to meet patients and build vital clinical experience

